# Metabolic bone disease in lion cubs at the London Zoo in 1889: the original animal model of rickets

**DOI:** 10.1186/1423-0127-17-S1-S36

**Published:** 2010-08-24

**Authors:** Russell W Chesney, Gail Hedberg

**Affiliations:** 1 Department of Pediatrics, The University of Tennessee Health Science Center, Memphis, Tennesee 38103, USA; 2 The San Francisco Zoological Gardens, San Francisco, California 94132, USA

## Abstract

In 1889 Dr. John Bland-Sutton, a prominent London surgeon, was consulted about fatal rickets in over 20 successive litters of lion cubs born at the London Zoo. He evaluated the diet and found the cause of rickets to be nutritional in origin. He recommended that goat meat with crushed bones and cod-liver oil be added to the lean horsemeat diet of the cubs and their mothers. Rickets were reversed, the cubs survived, and subsequent litters thrived. Thirty years later, in classic controlled studies conducted in puppies and young rats, the definitive role of calcium, phosphate and vitamin D in prevention and therapy of rickets was elucidated. Further studies led to identifying the structural features of vitamin D.

Although the Bland-Sutton diet provided calcium and phosphate from bones and vitamins A and D from cod-liver oil, some other benefits of this diet were not recognized. Taurine-conjugated bile salts, necessary for intestinal absorption of fat-soluble vitamins, were provided in the oil cold-pressed from cod liver. Unlike canine and rodent species, felines are unable to synthesize taurine, yet conjugate bile acids exclusively with taurine; hence, it must be provided in the diet. The now famous Bland-Sutton “experiment of nature,” fatal rickets in lion cubs, was cured by addition of minerals and vitamin D. Taurine-conjugated bile salts undoubtedly permitted absorption of vitamins A and D, thus preventing the occurrence of metabolic bone disease and rickets.

## Introduction

The story of the discovery of the cause and prevention of rickets is exciting and represents one of the most remarkable medical accomplishments of the 20^th^ century [1]. The prevalence of rickets in children living at northern latitudes was staggering, as high as 60% to 80% in some areas [1-4]. A body of work over a 15-year period beginning in 1917 defined the biologic properties of a “substance” in cod liver oil that was antirachitic. This substance also appeared to be produced by ultraviolet irradiation of the skin [1,4]. The structure of this substance, vitamin D, was determined and resulted in a Nobel Prize for Dr. Adolph Windaus in 1928 [5]. Historically, the addition of irradiated ergosterol to milk at 400 IU per liter, done independently by Steenbock and Hess [6-8] led to the virtual elimination of rickets in humans. The recognition that vitamin D is a group of prohormones that are converted to the active form (1,25(OH)_2_ D, or calcitriol) by a tightly regulated synthetic pathway is now recognized and accepted [9].

## Relevance of animal models

Studying proper animal models provides an understanding of vitamin D action. The utility of animal models was obvious to basic and clinical science scholars of rickets because of the overwhelming prevalence of the disease [1,2,4,10,11]. Although overt rickets was more frequently seen in lower socio-economic groups and in crowded tenements, it was common at all levels of society. Surveys showed that fully 60% to 80% of children who lived in urban areas in northern Europe during the period of urban migration and industrialization had clinically obvious rickets. Skies were blackened by the smog from burning coal, and diets were often inadequate in calcium, phosphate and vitamin D content. By the mid-1920's, there were many posited theories as to the cause of rickets, well summarized by Edwards Park in his 1923 review [4]. However, the need for carefully designed animal studies was obvious.

A somewhat obscure early model arose from the attempt to reverse rickets in lion cubs at the London Zoo in Regent’s Park. While this open label, non-controlled interventional trial reversed fatal rickets in cubs, its essential value was encouraging other investigators to design studies that explored the role of nutrients in the pathogenesis and treatment of rickets and osteomalacia [1,4].

It was from the studies of two groups (first, Edward Mellanby and co-workers in England, who evaluated the role of diet on the etiology of rickets in puppies, and second, the collaboration of Elmer V. McCollum, John Howland, Edwards A. Park and their team in Baltimore, MD, who studied rats fed restricted diets) that it was discerned that certain fats (cod-liver oil, butter and milk) could reverse the bone histologic and clinical features of rickets [2,4,9]. A substance in fat, far more concentrated in cod-liver oil than in butter, was the “antirachitic” factor. McCollum, who ultimately “discovered” vitamin D, was able to show that oxidized cod-liver oil (oxidation destroys vitamin A) still retained this antirachitic factor.

## Impact of the Bland-Sutton study

To fully appreciate why the studies of Mellanby and McCollum led to the discovery of vitamin D, a more probing analysis of Bland-Sutton's work with lion cubs is appropriate.

Three decades prior to Mellanby’s studies, in 1889, an “experiment of nature” occurred in the confines of the Zoological Garden in London. With the exception of a single litter, more than 20 consecutive litters of lion cubs perished. The surviving cubs were suckled by their dam for only two weeks, as she did not produce enough milk. The cubs were weaned very early onto lean horsemeat. They “invariably” developed extreme rickets and died [4]. Dr. John Bland-Sutton, a major figure in British surgery, was consulted and recommended a twice-weekly diet of goat flesh, goat bones and milk for both dams and cubs. He also suggested that the cubs lick cod-liver oil. He chose goat bones because they are softer than those of horses. Oddly, although Bland-Sutton wrote extensively, including his autobiography, he never recounted his experience in the zoo in a full publication. The curative diet was never formally described. There exist some conflicts in accounts of the exact diets fed the dams and cubs [4]. The message that Mellanby took away from Bland-Sutton’s work was that the curing of these lion cubs was related to the addition of fat to the diet. Mellanby used these ideas to design his famous dog studies in 1919 [12,13] and demonstrated the value of dietary fat, and in particular of cod-liver oil, in the prevention and treatment of rickets. Hence, the earlier studies of Bland-Sutton gained special importance. In contrast, Park, of the Baltimore team, felt that the true worth of Bland-Sutton’s work was somewhat overblown. He stated, “The so-called experiments of Bland-Sutton have had an influence which apparently they have not merited” [4]. In addition, Park pointed out that sometimes lions upon maturation “presented remarkable rickety changes in their skulls.” Despite Park’s reservations, numerous investigators of vitamin D deficiency in addition to Mellanby [2,4,9] recognized that the lion cubs in the London Zoo represented the initial animal experimental model of rickets.

Although cod-liver oil was curative of rickets in puppies and rats, the story in lion cubs is more complex and one in which numerous other factors are only now being recognized. An obvious interpretation of Bland-Sutton's dietary manipulations is that the cubs were being provided with minerals (calcium and phosphorus) from the goat bones and vitamin D from the cod-liver oil. The initial diet was lean horsemeat, which has an inverse ratio of calcium and phosphorus compared to bone and is devoid of fat. The limited milk intake, which was the main source of calcium and phosphorus in proper ratios, no doubt contributed to the under-mineralization of bones in the cubs [2]. Thus, lactation failure on the part of the dam was also a huge component of the development of rickets [4].

Several other factors, however, are critical to the diet of big cats such as lions, tigers and leopards [14]. Cats do not synthesize vitamin D_3_ adequately in the skin, and require a dietary source [15-18]. Fat-soluble vitamins work synergistically as well as antagonistically, particularly if imbalanced [19]. Felines also lack the ability to convert provitamin carotenoids, including ß-carotene, into active vitamin A [20]. Vitamin A is important to the integrity of the epithelium of the respiratory and digestive tracts. Large cats that lack vitamin A can develop sinusitis, diarrhea, blindness, conjunctivitis and neurologic signs [14]. Clinical signs from vitamin A-deficient immature lions include incoordination, "star gazing", blindness and intermittent convulsions [21]. Neurologic dysfunction accompanied by malformation of both the skull and the cervical vertebrae has been described in lions kept in captivity worldwide, and this dysfunction and malformation were most often related to vitamin A deficiency [22].

When big cats eat whole animals or are given bones they ingest calcium and phosphorus in a ratio of two parts calcium to one part phosphorous. Chunk lean meat has a calcium:phosphorus ratio of 1:15 to 1:30. Great cats deficient in calcium intake can develop osteomalacia; thus, their diets should be fortified with calcium [14]. It easier for cubs to chew softer bones, such as those of goats, rather than the hard bones of horses [4].

Cod-liver oil is replete with vitamin A and D, with a conventional value of 4000 to 5000 IU of A and 400 to 500 IU of D per teaspoonful (5 ml) [23]. However, in order to be utilized these fat-soluble vitamins must be absorbed from the intestine. Bile salts are essential in this biologic phenomenon. Cod-liver oil contains bile acids and, indeed, some bile salts. The conjugation of bile acids, especially those in herring [24] and cod [25], and in great cats, is dependent on the availability of taurine [23,24]. Because of the manner in which cod-liver oil is processed (extracted from fish liver at 82 °F under steam and then cold pressed to prevent emulsification), both taurine and bile acids are preserved [26]. This supplement (or medication) contains not only the vitamins but also the compounds that break down lipid micelles and permit intestinal fat absorption [26,27]. Moreover, cod, as with most marine fish or elasmobranchs, use taurine as an osmolyte, which is essential for cell volume regulation [27,28]. Typical supermarket cod contains 90 mg taurine per 100 g of raw fish [29]. The primary bile acids of cod, as with most fish, are cholic acid and to a lesser degree chenodeoxycholic acid [30].

The great cats, similar to their domestic cousins, are obligate carnivores. Felines have limited amounts of cysteinesulfinic acid decarboxylase, a rate-limiting enzyme in the synthesis of taurine from methionine and cysteine, hence taurine is an essential amino acid in cats. Cats fed a vegetable protein diet require taurine to prevent retinal degeneration and blindness [31,32]. A recent study has shown that the chunk horsemeat diet of exotic felines contains low taurine concentrations [33].

Cholic acid also is by far the dominant bile acid of lions [34]. Taurine conjugation of bile acids is mandatory, and glycine conjugation does not occur in either cod or lions [27,30,32,34]. Therefore, by providing cod-liver oil to the lion cubs, Bland-Sutton also increased their dietary intake of bile acids and taurine. This promoted absorption of fat-soluble vitamins [2,9].

Of interest, the goat can synthesize taurine, has a brisk enterohepatic circulation of bile acids, and its muscles contain ample taurine [35]. Thus, goat flesh was also a good source of taurine. Also, taurine is the most abundant free amino acid in goat milk [36].

The story of the elucidation of the pathogenesis of rickets obviously is also about the photocutaneous synthesis of vitamin D [1-4,9]. The remarkable observations of Palm [37] concerning the increased prevalence of rickets at northern latitudes opened up an entire line of rickets research and the role of ultraviolet light. Lions, with their thick fur, are far more dependent upon dietary sources of vitamin D. Because reversal of rickets came following the addition of fat to the lion cubs' diets, many doctors interpreted the Bland-Sutton experiments as showing the value of adding fat to a protein or milk diet in children [4,9]. Mellanby [12,13] understood the importance of studying controlled diets and was influenced by the rachitic cub story. McCollum recounts the lessons from the lion cubs in his classic textbook,* A History of Nutrition *[38]. But other skeptics were somewhat dismissive of Bland-Sutton's work. In Glasgow, another theory was promulgated. Findlay, Paton and Watson of the Glasgow school created a rickets model in puppies and felt that confinement and a lack of exercise, rather than diet, led to rickets [39]. Paton and Watson [40] later conceded that a poor diet, similar to that described by Mellanby, was needed to produce rickets. Findlay, in conversation with Bland-Sutton, commented that the surgeon also showed that goat meat and bones were needed for adult lions as well as cubs.

What became of John Bland-Sutton, esteemed surgeon and zoo consultant? He was the son of a tanner and cattle fattener who grew up on a small farm in London. He knew a great deal about and had a genuine interest in animals. Far from being a stuffy academician, he spoke with an occasionally embellished Cockney accent. He was also a close friend of Rudyard Kipling. He was knighted for his pioneering surgical work (especially for gynecological and gall bladder operations) and his service to the royal family. Ironically enough, Bland-Sutton was one of the first surgeons to perform a parathyroidectomy [41]. He was named Chief of Surgery at the Middlesex Hospital, near the London Zoological Garden in Regents Park, where the lion cubs were dying. Undoubtedly he was familiar with old wives' tales and folklore about the use of cod-liver oil for rickets. His experimental animal model of rickets, the quintessential calcium – vitamin D endocrine disorder, tweaked the interest of Mellanby. Bland-Sutton lived until 1936, long after vitamin D was added to milk.

Mellanby wisely chose puppies, and McCollum, another farm boy, chose rats, which were far easier to study. In trying to save rachitic lion cubs, Bland-Sutton had run into a species that needed to gnaw bones and had poor synthetic capacity for taurine, and for whom taurine is an essential amino acid. Cod-liver oil provided both the necessary vitamins and the taurine required for conjugating bile acids for intestinal fat-soluble vitamin absorption. Bland-Sutton was fortunate in his choice of dietary supplements.

## Competing interests

The authors declare that they have no competing interests.

## References

[B1] Rajakumar K Vitamin D, cod-liver oil, sunlight, and rickets: a historical perspective Pediatrics 2003 112 2 e132 135 10.1542/peds.112.2.e132 12897318

[B2] Holick MF Vitamin D deficiency N Engl J Med 2007 357 3 266 281 10.1056/NEJMra070553 17634462

[B3] Holick MF Chen TC Lu Z Sauter E Vitamin D and skin physiology: a D-lightful story J Bone Miner Res 2007 22 Suppl 2 V28 33 10.1359/jbmr.07s211 18290718

[B4] Park EA The etiology of rickets Physiol Rev 1923 3 106 163

[B5] Adolf Windaus - The Nobel Prize in Chemistry 1928. Nobel Lectures, Chemistry 1922-1941 1966 Amsterdam: Elsevier Publishing Company

[B6] Hess AF Weinstock M Antirachitic properties imparted to inert fluids and to green vegetables by ultraviolet irradiation J Biol Chem 1924 62 301 313

[B7] Steenbock H Black A Fat soluble vitamins XVII. The induction of growth-promoting and calcifying properties in a ration by exposure to ultraviolet light. J Biol Chem 1924 61 405 422 10.1126/science.60.1549.224 17773313

[B8] Steenbock H Black A Fat soluble vitamins XXIII. The induction of growth-promoting and calcifying properties in fats and their unsaponifiable constituents by exposure to light. J Biol Chem 1925 64 263 298

[B9] DeLuca HF Overview of general physiologic features and functions of vitamin D Am J Clin Nutr 2004 80 6 Suppl 1689S 1696S 15585789 10.1093/ajcn/80.6.1689S

[B10] Garland CF Grant WB Mohr SB Gorham ED Garland FC What is the dose-response relationship between vitamin D and cancer risk? Nutr Rev 2007 65 8 Pt 2 S91 95 10.1111/j.1753-4887.2007.tb00349.x 17867379

[B11] Holick MF High prevalence of vitamin D inadequacy and implications for health Mayo Clin Proc 2006 81 3 353 373 10.4065/81.3.353 16529140

[B12] Mellanby E An experimental investigation on rickets Lancet 1919 1 407 412

[B13] Mellanby E Experimental rickets. Medical Research (G.B.), Special Report Series SRS-61 1 7816747083

[B14] Hines R Diet, Feeding and Nutritional Care of Tigers, Lions and Leopards. http://www.2ndchance.info/bigcatdiet.htm accessed April 2008

[B15] How KL Hazewinkel HA Mol JA Dietary vitamin D dependence of cat and dog due to inadequate cutaneous synthesis of vitamin D Gen Comp Endocrinol 1994 96 1 12 18 10.1006/gcen.1994.1154 7843559

[B16] How KL Hazewinkel HA Mol JA Photosynthesis of vitamin D3 in cats Vet Rec 1994 134 15 384 8009805 10.1136/vr.134.15.384

[B17] Morris JG Ineffective vitamin D synthesis in cats is reversed by an inhibitor of 7-dehydrocholestrol-delta7-reductase J Nutr 1999 129 4 903 908 10203568 10.1093/jn/129.4.903

[B18] Morris JG Earle KE Anderson PA Plasma 25-hydroxyvitamin D in growing kittens is related to dietary intake of cholecalciferol J Nutr 1999 129 4 909 912 10203569 10.1093/jn/129.4.909

[B19] Bechert U Mortenson J Dierenfeld ES Cheeke P Keller M Holick M Chen TC Rogers Q Diet composition and blood values of captive cheetahs (Acinonyx jubatus) fed either supplemented meat or commercial food preparations J Zoo Wildl Med 2002 33 1 16 28 12216789 10.1638/1042-7260(2002)033[0016:DCABVO]2.0.CO;2

[B20] Schweigert FJ Raila J Wichert B Kienzle E Cats absorb beta-carotene, but it is not converted to vitamin A J Nutr 2002 132 6 Suppl 2 1610S 1612S 12042471 10.1093/jn/132.6.1610s

[B21] Bartsch RC Imes GD Jr. Smit JP Vitamin A deficiency in the captive African lion cub Panthera leo (Linnaeus 1758) Onderstepoort J Vet Res 1975 42 2 43 54 1208042

[B22] Shamir MH Shilo Y Fridman A Chai O Reifen R Miara L Sub-occipital craniectomy in a lion (Panthera leo) with occipital bone malformation and hypovitaminosis A J Zoo Wildl Med 2008 39 3 455 459 10.1638/2006-0064.1 18817011

[B23] Sullivan K Cod liver oil: the number one superfood. Wise Traditions in Food, Farming and the Healing Arts http://www.westonaprice.org/basicnutrition/codliveroil.html Accessed May 2008

[B24] Hedberg GE Dunker F Chesney RW Pathogenesis of metabolic bone disease in captive polar bears (Ursus maritimus). Polar Bears International 2007 http://www.polarbearsinternational.org/pbhc/hedberg.htm

[B25] Jones NR Taurine in fresh and iced skeletal muscle of codling (Gadus callarias) Biochem J 1953 56 324th Meeting xxii 13140246

[B26] Oleum morrhuae (U.S.P.) — Cod-liver oil. Henriette's Herbal Homepage http://www.henriettesherbal.com/eclectic/kings/gadus_oleu.html Accessed May 2008

[B27] Chesney RW Hedberg GE Rogers QR Dierenfeld ES Hollis BE Derocher A Does taurine deficiency cause metabolic bone disease and rickets in polar bear cubs raised in captivity? Adv Exp Med Biol 2009 643 325 331 full_text 19239163 10.1007/978-0-387-75681-3_33

[B28] Han X Patters AB Jones DP Zelikovic I Chesney RW The taurine transporter: mechanisms of regulation Acta Physiol (Oxf) 2006 187 12 61 73 10.1111/j.1748-1716.2006.01573.x 16734743

[B29] Gormley R Fish as a functional food. Ashtown Food Research Centre http://www.irishscientist.ie Accessed May 2008

[B30] Haslewood GA Recent developments in our knowledge of bile salts Physiol Rev 1955 35 1 178 196 14356932 10.1152/physrev.1955.35.1.178

[B31] Hayes KC Carey RE Schmidt SY Retinal degeneration associated with taurine deficiency in the cat Science 1975 188 4191 949 951 10.1126/science.1138364 1138364

[B32] Sturman JA Taurine in development Physiol Rev 1993 73 1 119 147 8419963 10.1152/physrev.1993.73.1.119

[B33] Hedberg GE Dierenfeld ES Rogers QR Taurine and zoo felids: considerations of dietary and biological tissue concentrations Zoo Biology 2007 26 6 517 531 10.1002/zoo.20158 19360598

[B34] Hagey LR Crombie DL Espinosa E Carey MC Igimi H Hofmann AF Ursodeoxycholic acid in the Ursidae: biliary bile acids of bears pandas, and related carnivores J Lipid Res 1993 34 11 1911 1917 8263415

[B35] Halais C Moir RJ Enterohepatic circulation of sulphur in female goats Br J Nutr 1990 63 2 239 248 10.1079/BJN19900111 2334662

[B36] Tripaldi C Martillotti F Terramoccia S Content of taurine and other free amino acids in milk of goats bred in Italy Small Rumin Res 1998 30 127 138 10.1016/S0921-4488(98)00095-9

[B37] Palm TA The geographical distribution and aetiology of rickets Practitioner 1890 270 321

[B38] McCollum EV A History of Nutrition. 1957 Cambridge, MA: Riverside Press

[B39] Findlay L The etiology of rickets: a clinical and experimental study Brit Med J 1908 2 13 10.1136/bmj.2.2479.13 PMC2436900 20763942

[B40] Paton DN Watson A Etiology of rickets; an experimental investigation Br Med J 1921 1 594A 10.1136/bmj.1.3147.594 PMC2414980 20770268

[B41] Delbridge LW Palazzo FF First parathyroid surgeon: Sir John Bland-Sutton and the parathyroids ANZ J Surg 2007 77 12 1058 1061 10.1111/j.1445-2197.2007.04324.x 17973666

